# One-Step Solvothermal Synthesis of Carbon Dots for Rapid and Accurate Determination of Hemin Content

**DOI:** 10.3390/molecules30061343

**Published:** 2025-03-17

**Authors:** Yiaobo Zhang, Lin Liu, Jiahui He, Chengzhi Huang, Lei Zhan, Chunmei Li

**Affiliations:** 1 Key Laboratory of Luminescence Analysis and Molecular Sensing (Southwest University), Ministry of Education, College of Pharmaceutical Sciences, Southwest University, Chongqing 400715, China; meowrry02@email.swu.edu.cn (Y.Z.); liul0077@163.com (L.L.); 13688192100@163.com (J.H.); chengzhi@swu.edu.cn (C.H.); 2 NMPA Key Laboratory for Quality Monitoring of Narcotic Drugs and Psychotropic Substance, Chongqing 401121, China

**Keywords:** carbon dots, hemin, solvothermal method, fluorescent inner filter effect

## Abstract

The development of sensitive and specific methods for the high-quality analysis of hemin-related drugs is significant in the pharmaceutical field. In this work, a simple and rapid method based on the fluorescent properties of carbon dots (CDs) was established for the determination of hemin in drugs. By taking melamine and ethylenediamine as the reaction materials, the fluorescent CDs were synthesized by a one-step solvothermal method, which can be used for the determination of hemin in drugs by the fluorescent inner filter effect. The as-prepared fluorescent CDs with rich functional groups on the surface displayed good water solubility, strong salt resistance, robust pH stability, and photobleaching resistance. Most importantly, the fluorescent excitation wavelength of fluorescent CDs was very close to the absorption wavelength of hemin, providing the evidence for the fluorescent inner filter effect. When the hemin concentration was in the range of 0.01–1 μM, there was a good linear relationship between the hemin content with the fluorescence intensity of CDs. The linear regression equation was (1 − *F*/*F*_0_) = 0.0897*c* + 0.0124, with a correlation coefficient (*R*^2^) of 0.9982 and a detection limit of 9 nM. This assay was successfully used to determine the content of hemin in the tablet, which displayed 97.9–105.5% of the labelled amount, with a relative standard deviation of less than 3%. The developed fluorescence method for the detection of hemin content displays the advantages of accurate, rapid, and high sensitivity, which could prove to be a useful tool for the determination of hemin supplement tablets.

## 1. Introduction

Hemin, a natural complex of protoporphyrin IX with an iron ion, is usually derived from degraded blood proteins. In vivo, hemin not only carries oxygen through the blood to maintain normal physiological functions, but also acts as an oxidation reduction catalyst to achieve the electron transfer process of Fe (III)/Fe (II) reversible oxidation reduction reactions [1]. Based on its oxidation reduction property, hemin is widely applied in natural iron supplements to cure iron deficiency anemia in the pharmaceutical field [2]. Back in the 1970s, hemin was approved by the Food and Drug Administration (FDA) for the treatment of acute porphyria [3]. In recent years, a combination of hemin and metformin has been investigated for the treatment of triple-negative breast cancer [4]. In addition to the pharmaceutical field, hemin and its derivatives have been extensively used in food [5], chemistry [6], biological science [7], and many other fields. As a consequence, the detection of hemin is exceedingly crucial for the high-quality evaluation of relative products.

At present, hemin analysis methods include electrochemistry [8], chemiluminescence [9], surface-enhanced Raman scattering [10], spectrophotometry [11], fluorescence [12], high-performance liquid chromatography [13], and mass spectrometry [14], etc. However, most of them are limited by some respective drawbacks, such as laborious operation, expensive equipment, and poor sensitivity. Relatively speaking, benefiting from simple operation, low cost, and high sensitivity, the fluorescence assay acts a pivotal part in constructing a fast, simple, economical, and sensitive detection method. For example, Zhang et al. constructed a fluorescent sensor assisted by human serum albumin for the rapid detection of hemin [15]. However, this method was restricted to the stability of human serum albumin. Therefore, it is still necessary to develop a simple, stable, and sensitive fluorescent sensor for the analysis of hemin.

Recently, fluorescent nanomaterials have been extensively investigated, such as organic fluorophores (OFs) [16], carbon dots (CDs) [17], quantum dots (QDs) [18], metal organic frames (MOF) [19], and metal nanoclusters (MNCs) [20]. However, some inherent defects restrict their further application. For example, OFs are prone to photobleaching [21], QDs and MNCs are potentially polluting the environment [22], and the fluorescence properties of MOFs are easily affected by the environment [23]. As a monodispersive carbon-based fluorescent nanomaterial, CDs have drawn tremendous attention due to their excellent fluorescence [24], outstanding catalytic performance [25], robust stability [26], favorable biocompatibility [27], simple preparation [28], and easy availability of materials [29]. By virtue of these distinct advantages, CDs have been widely applied in various fields such as chemical analysis [30], environmental monitoring [31], catalyst preparation [32], and medical imaging [33]. Notably, heteroatom-doped CDs enable pH-responsive biointerfaces for real-time cellular tracking via Förster resonance energy transfer mechanisms [34]. Additionally, their tunable red/near-infrared emission with two-photon absorption properties expands applications in deep-tissue imaging and photodynamic therapy [35]. For example, Qu et al. developed a CD-based ratiometric fluorescence method for the rapid on-site detection of phosgene [36]. Wu et al. prepared lysosome-targeted green fluorescent CDs for the intracellular detection and imaging of glutathione [37].

Until now, many synthesis methods have been developed to fabricate CDs, including top-down and bottom-up approaches [38]. Arc discharge [39], the laser ablation method [40], and electrochemical oxidation [41] are common top-down approaches, while combustion [42], solvothermal synthesis [43], chemical oxidation [44], template method [45], and microwave synthesis [46] are bottom-up approaches. However, the tough synthesis conditions and the requirement of laboratory apparatus with high energy consumption are the most crucial challenges to the employment of these methods. Therein, the solvothermal method is a kind of synthesis method with a simply controllable process, low cost, and green environmental protection, which is extensively used to prepare CDs with excellent optical properties, uniform particle size, and great water solubility [47]. For instance, Huang et al. prepared mitochondria-targeting CDs with homogeneous size, excellent photostability, and favorable biocompatibility via a one-step solvothermal treatment for peroxynitrite imaging in live cells [48]. Wu et al. obtained ultrabright CDs with photoluminescence quantum yields of 78% for rapid and accurate discrimination between live and dead cells [49].

The sensing principle is very important for the development of CD sensors. In general, the detection mechanism of fluorescence CDs mainly includes fluorescence resonance energy transfer (FRET) [50], the inner filter effect (IFE) [51], photoinduced electron transfer (PET) [52], static quenching [53], dynamic quenching [54], aggregation-induced emission [55], intramolecular charge transfer [56], hydrogen-bonding-induced emission [57], etc. By comparison, IFE requires neither a covalent connection between the receptor and the fluorophore nor the surface modification of fluorescent nanomaterials, which brings considerable flexibility and simplicity to analytical sensing. In the past, IFE was thought to be the source of errors in fluorescence analysis, which negatively influenced the fluorescence responses of analytes [58]. Later, it was found that the absorption signal of the analytes could be converted into a fluorescence signal by IFE, thus greatly improving the sensitivity of the analytical method. In light of its distinct performance, IFE has become an effective tool for developing simple, economical, and sensitive fluorescent sensors. For example, Dai et al. developed dual-emissive CDs for the determination of acid red 18 in drinks and candy using IFE [59]. Additionally, Li et al. quantified uric acid based on co-doped CDs via IFE [51].

**Scheme 1:**
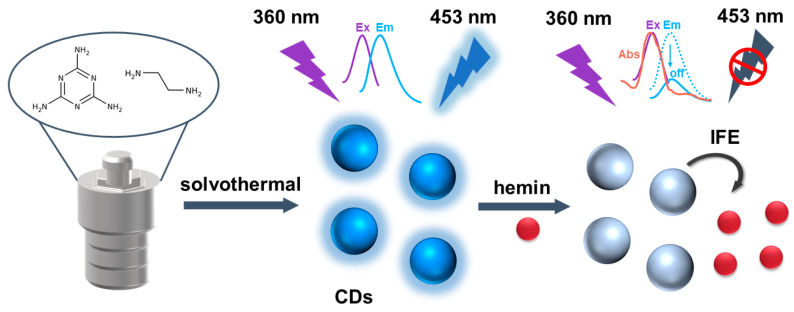
Illustration of fluorescence assays for hemin based on CDs.

Herein, we synthesized blue fluorescent CDs with excellent optical properties under a UV lamp via the one-step solvothermal method with ethylenediamine and melamine as carbon sources at 220 °C for 6 h. The prepared CDs had uniform particle size, good dispersion, and strong hydrophilicity with hydrophilic groups such as hydroxyl and carboxyl groups on the surface. In this experiment, the fluorescence excitation spectrum of CDs overlapped with the absorption spectrum of hemin to a large extent, which met the conditions of fluorescent IFE (Scheme 1). In the absence of hemin, the CDs emitted blue fluorescence under 360 nm excitation. In the presence of hemin, the hemin induced the fluorescence quenching of the CDs via IFE. Compared with the traditional analysis methods based on PET, FRET, and other strategies, our method based on fluorescent IFE has the advantages of being simple and fast without complex modifications to the fluorescence donor or covalent connection between the fluorescence donor and the absorption receptor. Therefore, a simple and rapid fluorescence analysis method was established based on the IFE for the detection of hemin.

## 2. Results and Discussion

### 2.1. Characterization of CDs

**Figure 1:**
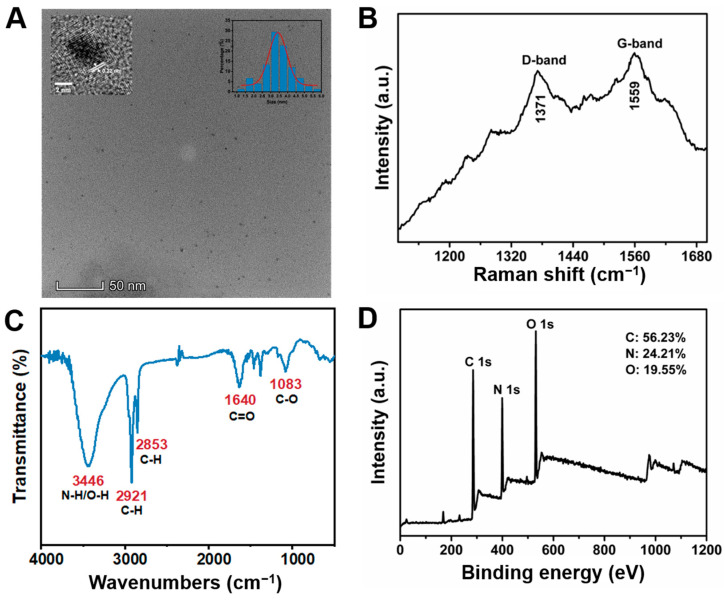
Characterization of CDs. (**A**) The high-resolution TEM image of CDs (insets were lattice spacings and particle size distribution of CDs). (**B**) The Raman spectrum of CDs. (**C**) The FTIR spectrum of CDs with functional group labeling. (**D**) The full-scale XPS spectrum of CDs.

Melamine and ethylenediamine were mixed in a high-pressure reactor under different temperatures and times to prepare blue-emitting CDs. As a result, the CDs that synthesized at 220 °C for 6 h displayed the highest fluorescence intensity (Figure S1). According to the optimized conditions, the CDs were characterized by varieties of techniques to acquire the properties. First, the morphology of CDs was investigated by a transmission electron microscope (TEM), which revealed that the CDs were uniformly distributed with a quasi-spherical shape in the aqueous solution (Figure 1A). Through the random statistics of 100 particles, the average particle size of the CDs was about 3.44 nm. Moreover, the CDs had an obvious lattice spacing of 0.22 nm agreed with the (100) facet of graphite [60,61]. Then, the Raman spectrum exhibited the sp^3^ configuration (D band) at 1371 cm^−1^ and the sp^2^ configuration (G band) at 1557 cm^−1^, which were in line with the disordered and graphitic structures, respectively (Figure 1B) [62,63]. In addition, the surface functional groups were characterized by Fourier transform infrared (FTIR) spectroscopy (Figure 1C). The results showed that a broad peak at 3446 cm^−1^ corresponded to N-H or O-H stretching vibrations, and peaks around 2921 cm^−1^ and 2853 cm^−1^ correspond to C-H stretching vibrations. The characteristic peaks at 1640 cm^−1^ and 1083 cm^−1^ suggested the existence of the C=O and C-O stretching vibrations. The X-ray photoelectron spectroscopy (XPS) was used to further analyze the surface chemical structure of CDs. The results indicated that the CDs consisted of carbon, nitrogen, and oxygen, which was consistent with the constituent elements in the materials. The relative atomic percentages on the surface were 56.23%, 24.21%, and 19.55%, respectively (Figure 1D). The high-resolution XPS spectrum of C 1s showed three kinds of bands: C-C/C=C (284.4 eV), C-O/C-N (285.2 eV), and C=O/C-N (287.3 eV). The O 1s spectrum revealed the presence of a C=O (530.4 eV) bond. The peak of N 1s was deconvoluted into two main peaks at 398.5 eV and 399.9 eV, which correspond to C-N and C=N. These XPS data revealed that the surface of CDs contained C-C, C-N, C=O, and C=N bonds (Figure S2), which was consistent with the FTIR experimental result.

**Figure 2:**
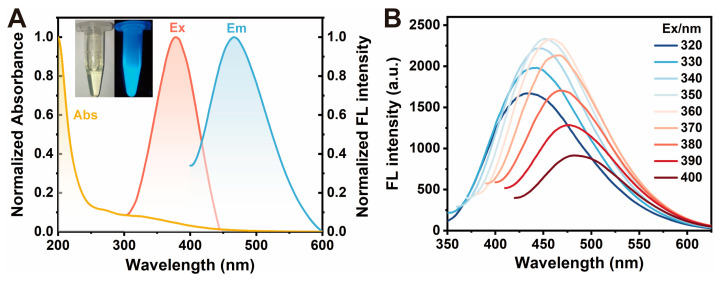
Optical properties of CDs. (**A**) UV-vis absorption and fluorescence spectra of the CDs. The yellow line is the UV-vis absorption of the CDs. The pink and blue lines are the excitation and emission spectra of CDs, respectively. The inset is the picture of CDs under a daylight lamp (**left**) and a 365 nm UV lamp (**right**). (**B**) Fluorescence emission spectra of CDs with increasing excitation wavelengths from 320 to 400 nm. Experimental conditions: CD concentration of 0.01 mg/mL, 10 mM PBS buffer (pH 7.4).

Furthermore, the UV-vis and fluorescence spectrum studies were performed to explore the optical properties of CDs (Figure 2A). Fluorescence spectra showed a maximum excitation peak at 378 nm and a maximum emission peak at 453 nm. Thus, the CDs exhibited blue fluorescence under a 365 nm UV lamp (Figure 2A inset). When the fluorescence excitation wavelength was increased from 320 nm to 400 nm, the emission showed an excitation-dependent behavior (Figure 2B). There are two mainly accepted fluorescence mechanisms to explain the excitation-dependent emission phenomenon of CDs: one is the size-dependent quantum confinement effect and the other is the surface state emission effect [64,65]. As shown in the inset of Figure 1A, the prepared CDs with a small distribution range of 2.5 to 5.5 nm were homogeneous enough, revealing that the size-dependent quantum confinement effect was not the primary reason for this excitation-dependent emission behavior [66]. In addition, the surface functional groups of CDs (such as hydroxyl and amino) probably acted as capture centers for excitons to form multiple emission sites or defect sites, resulting in the surface-state-dependent fluorescence of CDs [67]. As a consequence, the excitation-dependent behavior of CDs was mainly due to the influence of different surface states. In a word, using low-cost and easily available materials, we successfully synthesize CDs with rich water-soluble functional groups and excellent optical properties by using a simple solvothermal method.

### 2.2. Stability of CDs

**Figure 3:**
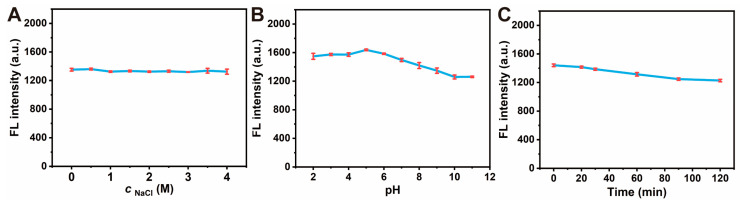
Stability test of CDs. (**A**) Salt resistance assay of CDs in different NaCl concentrations (0, 1, 2, 3, 4 M). (**B**) pH stability assay of CDs in different pH solutions (pH 2, 4, 6, 8, 12). (**C**) Photobleaching resistance assay of CDs for 2 h. Experimental conditions: CD concentration of 0.01 mg/mL, 10 mM PBS buffer (pH 7.4), 4 mM Britton–Robinson buffer (used to adjust pH).

When CDs are applied to analytical sensing in practical samples, it is essential to consider the optical stability in terms of salt resistance, pH stability, and photobleaching resistance. Therefore, the optical stability of the CDs was tested under assorted conditions. The fluorescence intensity remained almost constant when the NaCl concentrations were varied between 0 and 4 M, demonstrating the exceptional salt stability of CDs (Figure 3A). When the pH increased from 2 to 7, the fluorescence intensity of CDs remained relatively stable. However, it slightly decreased as the solution of CDs became alkaline (Figure 3B). This may be due to the fact that the surface of CDs was rich in hydroxyl, carboxyl, and other groups. When the pH increased, the surface of CDs could remove hydrogen ions and be negatively charged, which affected the fluorescence intensity [68]. As a whole, the pH stability could meet the requirements of the ordinary analytical environment. Lastly, the photobleaching resistance of CDs was examined under 360 nm excitation for 2 h. The fluorescence intensity hardly changed with the increase in time, which can be considered as the prepared CDs possessing fine photobleaching resistance (Figure 3C). The three stability tests manifested that the prepared CDs held robust stability and could be applied to the analysis and detection of complex environments.

### 2.3. Detection of Hemin by the CDs

**Figure 4:**
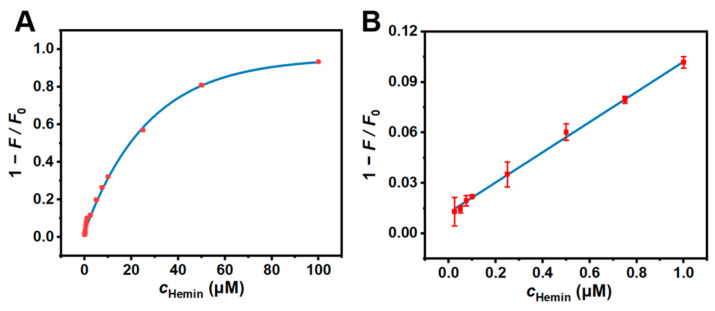
(**A**) The relationship between the fluorescence quenching efficiency (1 − *F*/*F*_0_) and the concentration of hemin over the range of 0.01, 0.025, 0.05, 0.075, 0.1, 0.25, 0.5, 0.75, 1, 2.5, 5, 7.5, 10, 25, 50, and 100 μM. (**B**) The linear plot between 1 − *F*/*F*_0_ and *c* over the range of 0.01–1 μM. Experimental conditions: CD concentration of 0.01 mg/mL, 10 mM PBS buffer (pH 7.4).

The CDs were allowed to be used as a probe for the analysis of hemin because of their excellent optical properties, good water solubility, and robust stability. Various levels of hemin from 0.01 to 100 μM were added to the sensing system (0.01 mg/mL CDs, PBS buffer, pH 7.4), and the responding fluorescence spectra were then recorded (Figure 4). With increasing concentrations of hemin, a decrease in fluorescence intensities at 453 nm was observed, indicating the quenching effect of hemin on the fluorescence emitted by the CDs. The quenching efficiency (1 − *F*/*F*_0_) displayed a good linear relationship in the linear range of 0.01–1 μM, with a correlation coefficient (*R*^2^) value of 0.9982. The obtained regression equation was 1 − *F/F*_0_ = 0.0897*c* + 0.0124. Meanwhile, the limit of detection (LOD = 3 *σ*/*k*) was calculated as 9 nM, indicating that the CDs could successfully provide a reliable platform for the quantification and analysis of hemin. In addition, our work shows comparative sensitivity towards hemin as compared to alternative methodologies that have been proposed (Table 1). All in all, these results attest to the sensitivity of our sensing system in detecting and quantifying hemin, foreseeing its potential utility in complex analytical applications.

### 2.4. Specificity of CDs

**Figure 5:**
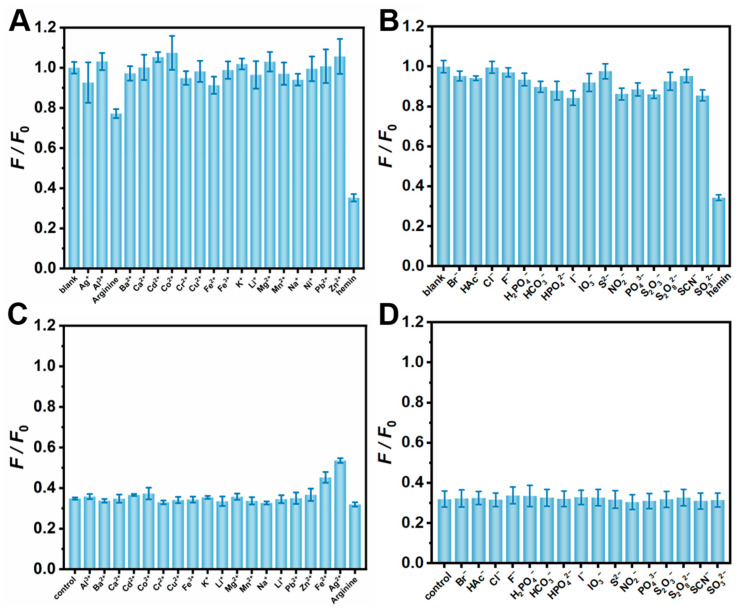
Selective experiments of CDs in different cations (**A**) and anions (**B**). Anti-interference experiments of CDs in different cations (**C**) and anions (**D**). Experimental conditions: CD concentration of 0.01 mg/mL, hemin concentration of 0.5 μM, ion concentration of 1 mM, 10 mM PBS buffer (pH 7.4).

Given that many metal cations and anions may affect the fluorescence intensity of fluorescent nanomaterials to some extent, the specificity study of CDs including selectivity and anti-interference ability was investigated in the environment of the different ions to check out the applicability of CDs for the detection of hemin. Sixteen common metal cations and anions were, respectively, selected to compare their effects on the fluorescence intensity of CDs in the experiments. As shown in Figure 5A,B, the fluorescence intensity ratio (*F*/*F*_0_) of CDs only decreased when hemin was added, while the fluorescence intensity ratio provided by other ions was consistent with that of the blank group, confirming the excellent selectivity of this detection method. The results of the anti-interference experiment clearly displayed that the response of the biosensor presented almost no change after the addition of the various interference analytes compared with the situation of only adding hemin, indicating that the CDs had high anti-interference to the detection of hemin (Figure 5C,D). This shows that the detection method had the desired selectivity and a robust anti-interference ability. In consequence, the method could monitor and analyze hemin in the complex system.

### 2.5. Mechanism Investigation of the Fluorescence Quenching of CDs by Hemin

**Figure 6:**
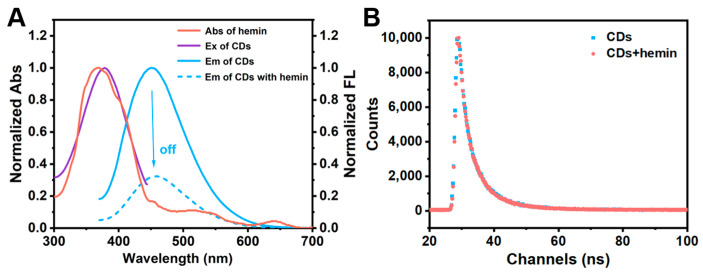
Quenching mechanism of CDs. (**A**) UV-vis absorption spectrum of hemin (red line), the fluorescence excitation spectrum of CDs (purple line), and the fluorescence emission spectrum of CDs in the absence (blue solid line) and presence (blue dotted line) of hemin. (**B**) The fluorescence lifetime of CDs in the absence (blue line) and presence (red line) of hemin. Experimental conditions: CD concentration of 0.01 mg/mL, hemin concentration of 0.1 μM, 10 mM PBS buffer (pH 7.4).

We further studied the mechanism for the fluorescence quenching of CD by hemin. The ultraviolet–visible absorption spectrum of hemin displayed a maximum absorption peak at 358 nm, which almost overlapped with the fluorescence excitation spectrum of CDs. This resulted in the weakened fluorescence emission intensity of CDs in the presence of hemin (Figure 6A). Therefore, we supposed that the detection mechanism may be IFE or FRET. The fluorescence lifetime of a fluorescent substance is related to its structure, the polarity and viscosity of its microenvironment, and other conditions [73,74,75]. Thus, the differences in the system can be directly inferred from fluorescence lifetime. That is to say, IFE does not change the fluorescence lifetime of the nanomaterial, while FRET will shorten the fluorescence lifetime, so the fluorescence lifetime of CDs before and after the addition of hemin were further measured. The results showed that the fluorescence lifetime of CDs was 5.75 ns and 5.74 ns in the absence and presence of hemin, respectively (Figure 6B and Table S1). It can be considered that hemin has no impact on the fluorescence lifetime of the CDs. According to the above results, it was confirmed that the quenching mechanism was the IFE rather than FRET [76].

### 2.6. Determination of Hemin in Real Samples

In the end, the proposed CDs were applied to assess the content of hemin in Changxing-brand hemin iron-supplement tablets. The reproducibility and accuracy of the method were evaluated by examining the results of the method and the recovery of the labeled amount. The recoveries of hemin in drugs were estimated to be 97.9–105.5%, with a relative standard deviation (RSD) of less than 3% (Table 2). The results showed that the sensor could detect hemin in the spiked samples with high precision and excellent reproducibility, indicating that it had improved practical applications.

## 3. Materials and Methods

### 3.1. Apparatus

The heat source during the synthesis of CDs was provided by an electric thermostatic drying oven (Shanghai Yiheng, Shanghai, China). The ultraviolet–visible spectra were carried out on a UV-3600 spectrophotometer (Shimadzu, Kyoto, Japan). The element composition of CDs was analyzed by an ESCALAB250 X-ray photoelectron spectrometer (Thermo Fisher Scientific, Waltham, MA, USA). The morphology, particle size, and lattice of CDs were characterized using a Tec-naiG2F20STWIN high-resolution transmission electron microscope (FEI Company, Portland, OR, USA). The infrared spectra were obtained using an FTIR-8400S Fourier transform infrared spectroscopy spectrometer (Shimadzu, Japan). Fluorescence spectra were recorded by an F-2500 fluorescence spectrophotometer (Hitachi, Tokyo, Japan). The fluorescence lifetime was performed by an FS-TCSPC Time-resolved Transient Fluorescence spectrometer (Horiba Jobin Yvon, Paris, France). Raman spectra of CDs in the solution of silver nanoparticle were scanned by a LabRAMHR800 laser confocal Raman spectrometer (Horiba Jobin Yvon, Paris, France).

### 3.2. Materials

Melamine and ethylenediamine were purchased from the Aladdin reagent company (Shanghai, China). Dimethyl sulfoxide was purchased from Chongqing Chuandong Chemical Co., Ltd. (Chongqing, China) as a solvent to dissolve the hemin. NaCl was purchased from Chuandong Chemical Co., Ltd. (Chongqing, China) and used for controlling the ionic strength. The water used in the experiments was ultrapure (18.2 MΩ·cm^−1^, Mili-Q, Merck KgaA, Darmstadt, Germany).

### 3.3. Synthesis of CDs

CDs were synthesized via a one-step solvothermal method. In total, 50 mg melamine, 200 μL ethylenediamine, and 5 mL ultrapure water were added to the polytetrafluoroethylene reactor, covered with a stainless steel jacket, and placed in an air blast drying oven. After the reaction at 220 °C for 6 h, the reactor was naturally cooled to room temperature to obtain a pale yellow liquid. The liquid was dialyzed against ultrapure water through a dialysis membrane (500–1000 DA) for 24 h. The purified CD solution was lyophilized, yielding light-brown solid products. The lyophilized CDs were weighed precisely and redispersed in ultrapure water to prepare a stock solution (1.0 mg/mL). Then, the stock solution was precisely diluted to 0.01 mg/mL, which was stored at 4 °C in the dark for further use.

### 3.4. Fluorescence Property and Stability Test

The excitation and emission spectra of CDs (0.01 mg/mL) were scanned on an F-2500 fluorescence spectrophotometer to determine their maximum excitation and emission wavelength (slit 10 nm, voltage 700 V). The salt stability of CDs was tested by recording the fluorescence intensity of CDs (0.01 mg/mL) in different concentrations of sodium chloride (NaCl) (0, 0.5, 1.0, 1.5, 2.0, 2.5, 3.0, 3.5, and 4.0 M). The pH stability was assessed by measuring the fluorescence intensity of CDs (0.01 mg/mL) in different pH solutions (2.21, 3.29, 4.10, 5.02, 6.09, 7.0, 7.96, 8.95, 9.91, and 10.88). In addition, photobleaching resistance was evaluated by detecting the fluorescence intensity of CDs (0.01 mg/mL) at different times (0, 15, 30, 60, 90, and 120 min).

### 3.5. Quantification of Hemin

The hemin was first dissolved with a small amount of dimethyl sulfoxide (DMSO) to prepare different concentrations of solution (0.01, 0.025, 0.05, 0.075, 0.1, 0.25, 0.5, 0.75, 1, 2.5, 5, 7.5, 10, 25, 50, and 100 μM). After mixing 50 μL CDs (0.01 mg/mL), 50 μL PBS, 50 μL hemin, and 350 μL pure water, the fluorescence intensity of CDs was detected. According to the relationship between the fluorescence intensity of CDs and the concentrations of hemin, the curve was fitted to establish the linear equation between the fluorescence intensity ratio (1 − F/F_0_) and the concentrations of hemin (c) (F and F_0_ were the fluorescence intensity with and without hemin, respectively).

### 3.6. Specificity of CDs

The selectivity experiment was investigated by comparing the fluorescence intensity of CDs (0.01 mg/mL) in the presence of different interfering ions (1 mM) and the hemin (0.5 μM). The anti-interference experiments were studied by measuring the fluorescence intensity of CDs (0.01 mg/mL) under different interfering ion conditions (1 mM) in the presence of hemin.

### 3.7. Fluorescence Lifetime of CDs

The fluorescence lifetime of CDs before and after the response to hemin was measured at room temperature by time-correlated single-photon counting (TCSPC) with the excitation and emission wavelength of 360 nm and 453 nm, respectively.

### 3.8. Detection of Hemin in Drugs

Three pieces of Changxing-brand hemin iron-supplemented tablets (0.6 g/piece) were comminuted and accurately weighed. Then, the powder was dissolved with DMSO and gradually diluted with water until the final concentration of hemin was 0.5 μM. Subsequently, the fluorescence spectra of mixtures (50 μL CDs, 50 μL PBS, 50 μL hemin, and 350 μL pure water) were recorded under the excitation of 360 nm. The blank group was an unmediated solution. Three parallel groups were measured three times each.

## 4. Conclusions

In summary, we developed blue fluorescent CDs via a simple one-step solvothermal method by reasonably using melamine and ethylenediamine as carbon sources. The as-prepared CDs presented good optical properties and water solubility and high stability, which contributed to analytical applications as excellent fluorescent probes. TEM, FTIR, XPS, Raman, and other characterizations proved that the CDs were quasi-spherical nanoparticles with massive water-soluble groups such as amino and carboxyl groups on the surface. Since the fluorescence excitation spectrum of CDs almost overlapped with the UV-vis absorption spectrum of hemin, a label-free fluorescence analysis method based on the IFE was successfully established for the determination of hemin with a detection limit of 9 nM. Furthermore, our methods had favorable selectivity and anti-interference, which was also applied in the determination of hemin content in drugs. This method is simple, fast, and highly selective, which provides a powerful tool for the detection of hemin, indicating a prospective application in drug quality analysis and biological sample analysis.

## Figures and Tables

**Table 1:** Comparison of the IFE-based CDs with other reported strategies for hemin detection.

Methods	Detection System	Linear Range	Detection Limit	Reference
Fluorescence	fluorescent signal reporter molecule	0.31–2.5 μM	9.84 nM	[15]
Fluorescence	AO-PS2.M/rGO	2.81–4.37 μM	50 nM	[69]
Fluorescence	Si-NPs	0.05–125 μM	29.5 nM	[70]
Chemiluminescence	artemisinin-luminol	0.2–10 μM	200 nM	[71]
Spectrophotometry	extraction	1.15–9.2 μM	72 nM	[72]
Fluorescence	CDs	0.01–1 μM	9 nM	This work

**Table 2:** Detection of hemin content in drugs.

Group	Labeled Amount (g/Piece)	Determined Amount (g/Piece)	Recovery (%)	RSD (*n* = 3, %)
1	0.6	0.587	97.9	1.6
2	0.6	0.592	98.7	2.8
3	0.6	0.633	105.5	2.0

Experimental conditions: CD concentration of 0.01 mg/mL, PBS buffer (pH 7.4). Three parallel groups were measured three times each.

## Data Availability

Data are contained within the article and Supplementary Materials.

## Supplementary Materials

The following supporting information can be downloaded at: https://www.mdpi.com/article/10.3390/molecules30061343/s1, Figure S1: Optimization of synthesis parameters of CDs; Figure S2: The high-resolution XPS spectrum of CDs; Table S1: The influence of hemin on the fluorescence lifetime of CDs.

## References

[1] Ryu W.H., Gittleson F.S., Thomsen J.M., Li J., Schwab M.J., Brudvig G.W., Taylor A.D. (2016). Heme biomolecule as redox mediator and oxygen shuttle for efficient charging of lithium-oxygen batteries. Nat. Commun..

[2] Estrada D.T., Schwartz K.A. (2007). Iron absorption: Comparison of oral heme-bound iron with inorganic ferrous sulfate. Blood.

[3] Peterson A., Bossenmaier I., Cardinal R., Watson C.J. (1976). Hematin treatment of acute porphyria: Early remission of an almost fatal relapse. JAMA.

[4] Lee J., Yesilkanal A.E., Wynne J.P., Frankenberger C., Liu J., Yan J., Elbaz M., Rabe D.C., Rustandy F.D., Tiwari P. (2019). Effective breast cancer combination therapy targeting BACH1 and mitochondrial metabolism. Nature.

[5] Whalin J., Wu Y.T., Wang Y.F., Suman S.P., Shohet J.L., Richards M.P. (2024). Use of plasma induced modification of biomolecules (PLIMB) to evaluate hemin dissociation from fish and bovine methemoglobins. Food Chem..

[6] Liang H., Li J., Zhang J., Peng W., Li J., Liu J. (2024). Tri-functional Fe-based electrocatalyst with sturdy three-dimensional frame construction for the ORR, OER and HER. J. Mater. Chem. A.

[7] Song N., Fan X., Guo X., Tang J., Li H., Tao R., Li F., Li J., Yang D., Yao C. (2024). A DNA/Upconversion Nanoparticle Complex Enables Controlled Co-Delivery of CRISPR-Cas9 and Photodynamic Agents for Synergistic Cancer Therapy. Adv. Mater..

[8] Tang S., Xie X., Li L., Zhou L., Xing Y., Chen Y., Cai K., Li F., Zhang J. (2024). High fidelity detection of miRNAs from complex physiological samples through electrochemical nanosensors empowered by proximity catalysis and magnetic separation. Biosens. Bioelectron..

[9] Cheng K., Wang H., Sun S., Wu M., Shen H., Chen K., Zhang Z., Li S., Lin H. (2023). Specific Chemiluminescence Imaging and Enhanced Photodynamic Therapy of Bacterial Infections by Hemin-Modified Carbon Dots. Small.

[10] Li D., Li C., Liang A., Jiang Z. (2019). SERS and fluorescence dual-mode sensing trace hemin and K^+^ based on G-quarplex/hemin DNAzyme catalytic amplification. Sens. Actuators B Chem..

[11] Wu N., Wong K.-Y., Yu X., Zhao J.-W., Zhang X.-Y., Wang J.-H., Yang T. (2024). Multispectral 3D DNA Machine Combined with Multimodal Machine Learning for Noninvasive Precise Diagnosis of Bladder Cancer. Anal. Chem..

[12] Wang F., Dong X., Zuo Y., Xie Z., Guan R. (2024). Effectively enhancing red fluorescence strategy and bioimaging applications of carbon dots. Mater. Today Phys..

[13] Isaji Y., Ogawa N.O., Takano Y., Ohkouchi N. (2020). Quantification and carbon and nitrogen isotopic measurements of heme B in environmental samples. Anal. Chem..

[14] Sikora K.N., Hardie J.M., Castellanos-García L.J., Liu Y., Reinhardt B.M., Farkas M.E., Rotello V.M., Vachet R.W. (2020). Dual mass spectrometric tissue imaging of nanocarrier distributions and their biochemical effects. Anal. Chem..

[15] Chao X., Yao D., Chen C., Zhang C. (2023). An efficient human serum albumin-assisted fluorescence approach for hemin detection. Environ. Technol. Innov..

[16] Matsushima T., Qin C., Teng T., Kamatham N., Sosa Vargas L., Kreher D., Heinrich B., Ishii T., Terakawa S., Leyden M.R. (2024). Efficient Electroluminescence from Organic Fluorophore-Containing Perovskite Films. Adv. Mater..

[17] Zheng G.-S., Shen C.-L., Niu C.-Y., Lou Q., Jiang T.-C., Li P.-F., Shi X.-J., Song R.-W., Deng Y., Lv C.-F. (2024). Photooxidation triggered ultralong afterglow in carbon nanodots. Nat. Commun..

[18] van Riggelen-Doelman F., Wang C.-A., de Snoo S.L., Lawrie W.I.L., Hendrickx N.W., Rimbach-Russ M., Sammak A., Scappucci G., Déprez C., Veldhorst M. (2024). Coherent spin qubit shuttling through germanium quantum dots. Nat. Commun..

[19] Desai A.V., Seymour V.R., Ettlinger R., Pramanik A., Manche A.G., Rainer D.N., Wheatley P.S., Griffin J.M., Morris R.E., Armstrong A.R. (2023). Azo-functionalised metal–organic framework for charge storage in sodium-ion batteries. Chem. Comm..

[20] Li H., Kang X., Zhu M. (2024). Superlattice Assembly for Empowering Metal Nanoclusters. Acc. Chem. Res..

[21] Jung S.-R., Deng Y., Kushmerick C., Asbury C.L., Hille B., Koh D.-S. (2018). Minimizing ATP depletion by oxygen scavengers for single-molecule fluorescence imaging in live cells. Proc. Natl. Acad. Sci. USA.

[22] Cao W., Liu X., Huang X., Liu Z., Cao X., Gao W., Tang B. (2022). Hepatotoxicity-related oxidative modifications of thioredoxin 1/peroxiredoxin 1 induced by different cadmium-based quantum dots. Anal. Chem..

[23] Moayed Mohseni M., Jouyandeh M., Mohammad Sajadi S., Hejna A., Habibzadeh S., Mohaddespour A., Rabiee N., Daneshgar H., Akhavan O., Asadnia M. (2022). Metal-organic frameworks (MOF) based heat transfer: A comprehensive review. Chem. Eng. J..

[24] Shi Y., Xia Y., Zhou M., Wang Y., Bao J., Zhang Y., Cheng J. (2024). Facile synthesis of Gd/Ru-doped fluorescent carbon dots for fluorescent/MR bimodal imaging and tumor therapy. J. Nanobiotechnol..

[25] Tang T., Zhao J., Shen Y., Yang F., Yao S., An C. (2024). Carbon dots bridged Zn0.5Cd0.5S with interfacial amide bond facilitating electron transfer for efficient photocatalytic hydrogen peroxide production. Appl. Catal. B-Environ. Energy.

[26] Ai L., Xiang W., Xiao J., Liu H., Yu J., Zhang L., Wu X., Qu X., Lu S. (2024). Tailored Fabrication of Full-Color Ultrastable Room-Temperature Phosphorescence Carbon Dots Composites with Unexpected Thermally Activated Delayed Fluorescence. Adv. Mater..

[27] Chen H., Geng X., Ning Q., Shi L., Zhang N., He S., Zhao M., Zhang J., Li Z., Shi J. (2024). Biophilic Positive Carbon Dot Exerts Antifungal Activity and Augments Corneal Permeation for Fungal Keratitis. Nano Lett..

[28] Zhang Y., Chen L., Liu B., Yu S., Yang Y., Liu X. (2024). Multicolor Afterglow Carbon Dots: Luminescence Regulation, Preparation, and Application. Adv. Funct. Mater..

[29] Ma H., Guan L., Chen M., Zhang Y., Wu Y., Liu Z., Wang D., Wang F., Li X. (2022). Synthesis and enhancement of carbon quantum dots from Mopan persimmons for Fe^3+^ sensing and anti-counterfeiting applications. Chem. Eng. J..

[30] Bai X., Deng W., Wang J., Zhou M. (2025). Enrichment-enhanced detection strategy in the optimized monitoring system of dopamine with carbon dots-based probe. Chin Chem. Lett..

[31] Guo P., He X., An X., Zhang X., Liang S., Zhang J., Fu Y. (2024). N-doped carbon dots/SnS_2_ heterostructures for ultra-fast and sub-ppb-level detection of NO_2_ in multiple environments. Chem. Eng. J..

[32] Zhou M., Xu S., Zhang W., Shi G., He Y., Qiao X., Pang X. (2024). How Luminescence Performances of Silicon-Doped Carbon Dots Contribute to Copper-Catalyzed photoATRP?. ACS Catal..

[33] Barhum H., McDonnell C., Peltek O., Jain R., Amer M., Kain D., Elad-Sfadia G., Athamna M., Blinder P., Ginzburg P. (2024). In-Brain Multiphoton Imaging of Vaterite Cargoes Loaded with Carbon Dots. Nano Lett..

[34] Tan N.K., Chan H., Lu Z., Zreiqat H., Lakhwani G., Lesani P., New E.J. (2024). Ultrasensitive Dual Fluorophore-Conjugated Carbon Dots for Intracellular pH Sensing in 3D Tumor Models. ACS Appl. Mater. Interfaces.

[35] Lesani P., Mohamad Hadi A.H., Lu Z., Palomba S., New E.J., Zreiqat H. (2021). Design principles and biological applications of red-emissive two-photon carbon dots. Commun. Mater..

[36] Zhang J., Li Y., Hu J., Sun Y., Yang R., Li Z., Qu L. (2023). Rapid colorimetric and ratiometric fluorescence method for on-site detection and visualization of phosgene by amino-functionalized carbon dot-based portable droplet system. Chem. Eng. J..

[37] Wang Z., Xu K.F., Wang G., Durrani S., Lin F., Wu F.G. (2023). “One stone, five birds”: Ultrabright and multifaceted carbon dots for precise cell imaging and glutathione detection. Chem. Eng. J..

[38] Chen B.B., Liu M.L., Li C.M., Huang C.Z. (2019). Fluorescent carbon dots functionalization. Adv. Colloid Interface Sci..

[39] Xu X., Ray R., Gu Y.l., Ploehn H.J., Gearheart L., Raker K., Scrivens W.A. (2004). Electrophoretic analysis and purification of fluorescent single-walled carbon nanotube fragments. J. Am. Chem. Soc..

[40] Li Q., Zhao H., Yang D., Meng S., Gu H., Xiao C., Li Y., Cheng D., Qu S., Zeng H. (2024). Direct in Situ Fabrication of Multicolor Afterglow Carbon Dot Patterns with Transparent and Traceless Features via Laser Direct Writing. Nano Lett..

[41] Liu M., Xu Y., Niu F., Gooding J.J., Liu J. (2016). Carbon quantum dots directly generated from electrochemical oxidation of graphite electrodes in alkaline alcohols and the applications for specific ferric ion detection and cell imaging. Analyst.

[42] Wu J.Y., Huang Y.C. (2024). Low-energy-consumption rapid synthesis of carbon dots at room temperature from combusted food waste with versatile analytical applications. Food Chem..

[43] Li J., Li W., Wang H., Zhao X., Gong X. (2024). Solid-state fluorescent carbon dots-based composite optical waveguide film enables transparent and high-performance luminescent solar concentrators. Appl. Energy.

[44] Bao L., Liu C., Zhang Z., Pang D. (2015). Photoluminescence-tunable carbon nanodots: Surface-state energy-gap tuning. Adv. Mater..

[45] Chernyak S., Podgornova A., Dorofeev S., Maksimov S., Maslakov K., Savilov S., Lunin V. (2020). Synthesis and modification of pristine and nitrogen-doped carbon dots by combining template pyrolysis and oxidation. Appl. Surf. Sci..

[46] Zhang D.-H., Yang L., Li N., Su K., Liu L., Li C.-Y. (2024). Detection of ciprofloxacin and pH by carbon dots and rapid, visual sensing analysis. Food Chem..

[47] Liu M.L., Chen B.B., Li C.M., Huang C.Z. (2019). Carbon dots: Synthesis, formation mechanism, fluorescence origin and sensing applications. Green Chem..

[48] Bai Y., Wang Y., Cao L.p., Jiang Y.j., Li Y.f., Zou H.y., Zhan L., Huang C.z. (2021). Self-targeting carbon quantum dots for peroxynitrite detection and imaging in live cells. Anal. Chem..

[49] Yu X., Liu X., Jiang Y., Li Y., Gao G., Zhu Y., Lin F., Wu F. (2022). Rose bengal-derived ultrabright sulfur-doped carbon dots for fast discrimination between live and dead cells. Anal. Chem..

[50] Zou M., Li Q., Ji X., Ding C. (2024). Low biofouling strategy for simultaneous determination of two proteins related to one tumor in human serum. Sens. Actuators B Chem..

[51] Li F., Chen J., Wen J., Peng Y., Tang X., Qiu P. (2022). Ratiometric fluorescence and colorimetric detection for uric acid using bifunctional carbon dots. Sens. Actuators B Chem..

[52] Jaiswal A., Rani S., Singh G.P., Archana T., Hassan M., Nasrin A., Gomes V.G., Saxena S., Shukla S. (2024). Rapid additive manufacturing of all-carbon, all-dielectric metastructures. Addit. Manuf..

[53] Liu M., Wei S., Xie Y., Su K., Yin X., Song X., Hu K., Sun G., Liu Y. (2025). Ratiometric fluorescence sensor based on chiral europium-doped carbon dots for specific and portable detection of tetracycline. Sens. Actuators B Chem..

[54] Kim S.E., Yoon J.C., Muthurasu A., Kim H.Y. (2024). Fluorescence immunoassay using triangular carbon dots for detection of the cardiac marker Troponin T in acute myocardial infarction. Sens. Actuators B Chem..

[55] Miao J., Yu J., Zhao X., Chen X., Zhu C., Cao X., Huang Y., Li B., Wu Y., Chen L. (2024). Molecular imprinting-based triple-emission ratiometric fluorescence sensor with aggregation-induced emission effect for visual detection of doxycycline. J. Hazard. Mater..

[56] Li X., Zhang G., Li N., Xu Q., Li H., Lu J., Chen D. (2024). Self-Floating Photocatalytic System for Highly Efficient Hydrogen Peroxide Production and Organic Synthesis on Carbon Dots Decorated Conjugated Microporous Polymer. Adv. Funct. Mater..

[57] Ai L., Xiang W., Li Z.-W., Liu H., Xiao J., Song H., Yu J., Song Z., Zhu K., Pan Z. (2024). Hydrogen Bond-Induced Flexible and Twisted Self-Assembly of Functionalized Carbon Dots with Customized-Color Circularly Polarized Luminescence. Angew. Chem. Int. Ed..

[58] Yuan P., Walt D.R. (1987). Calculation for fluorescence modulation by absorbing species and its application to measurements using optical fibers. Anal. Chem..

[59] Dai R., Hu Y. (2022). Green/red dual emissive carbon dots for ratiometric fluorescence detection of acid red 18 in food. Sens. Actuators B Chem..

[60] Wang Y., Li Z., Guo G., Xia Y. (2023). Liver injury traceability: Spatiotemporally monitoring oxidative stress processes by unit-emitting carbon dots. Anal. Chem..

[61] Su W., Tan M., Wang Z., Zhang J., Huang W., Song H., Wang X., Ran H., Gao Y., Nie G. (2023). Targeted degradation of PD-L1 and activation of the STING pathway by carbon-dot-based PROTACs for cancer immunotherapy. Angew. Chem. Int. Ed..

[62] Xu J., Yokota Y., Wong R.A., Kim Y., Einaga Y. (2020). Unusual electrochemical properties of low-doped boron-doped diamond electrodes containing sp^2^ carbon. J. Am. Chem. Soc..

[63] Picheau E., Impellizzeri A., Rybkovskiy D., Bayle M., Mevellec J.Y., Hof F., Saadaoui H., Noé L., Torres Dias A.C., Duvail J.L. (2021). Intense Raman D band without sisorder in flattened carbon nanotubes. ACS Nano.

[64] Ganguly S., Das P., Bose M., Mondal S., Das A.K., Das N.C. (2017). Strongly blue-luminescent N-doped carbogenic dots as a tracer metal sensing probe in aqueous medium and its potential activity towards in situ Ag-nanoparticle synthesis. Sens. Actuators B Chem..

[65] Costa A.I., Barata P.D., Moraes B., Prata J.V. (2022). Carbon dots from coffee grounds: Synthesis, characterization, and detection of noxious nitroanilin. Chemosensors.

[66] Sun D., Liu T., Wang C., Yang L., Yang S., Zhuo K. (2020). Hydrothermal synthesis of fluorescent carbon dots from gardenia fruit for sensitive on-off-on detection of Hg^2+^ and cysteine. Spectrochim. Acta A Mol. Biomol. Spectrosc..

[67] Ding H., Yu S., Wei J., Xiong H. (2016). Full-color light-emitting carbon dots with a surface-state-controlled luminescence mechanism. ACS Nano.

[68] Jin X., Sun X., Chen G., Ding L., Li Y., Liu Z., Wang Z., Pan W., Hu C., Wang J. (2015). pH-sensitive carbon dots for the visualization of regulation of intracellular pH inside living pathogenic fungal cells. Carbon.

[69] Shi Y., Huang W., Luo H., Li N. (2011). A label-free DNA reduced graphene oxide-based fluorescent sensor for highly sensitive and selective detection of hemin. Chem. Comm..

[70] Du N., Zhang H., Wang J., Dong X., Li J., Wang K., Guan R. (2022). Fluorescent silicon nanoparticle–based quantitative hemin assay. Anal. Bioanal. Chem..

[71] Gao W., Wang C., Muzyka K., Kitte S.A., Li J., Zhang W., Xu G. (2017). Artemisinin-luminol chemiluminescence for forensic bloodstain detection using a smart phone as a detector. Anal. Chem..

[72] Lombardo M.E., Araujo L.S., Ciccarelli A.B., Batlle A. (2005). A spectrophotometric method for estimating hemin in biological systems. Anal. Biochem..

[73] Michels L., Gorelova V., Harnvanichvech Y., Borst J.W., Albada B., Weijers D., Sprakel J. (2020). Complete microviscosity maps of living plant cells and tissues with a toolbox of targeting mechanoprobes. Proc. Natl. Acad. Sci. USA.

[74] Chen Z., Lu X., Qiao J., Liu J., Qin W. (2022). Mechanoresponsive spin via spin–lattice coupling in organic cocrystals. Nano Lett..

[75] Chung C.W., Stephens A.D., Ward E., Feng Y., Davis M.J., Kaminski C.F., Kaminski Schierle G.S. (2022). Label-free characterization of amyloids and alpha-synuclein polymorphs by exploiting their intrinsic fluorescence property. Anal. Chem..

[76] Chen S., Yu Y., Wang J. (2018). Inner filter effect-based fluorescent sensing systems: A review. Anal. Chim. Acta..

