# Supplementary material for: One-Step Solvothermal Synthesis of Carbon Dots for Rapid and Accurate Determination of Hemin Content
Source: Molecules. 2025 Mar 17;30(6):1343. doi: 10.3390/molecules30061343 (PMC11944978; doi:10.3390/molecules30061343)
Supplement: Supplementary file 1 [file molecules-30-01343-s001.zip › molecules-3479258-supplementary.pdf]

*Supplementary Materials*

# One-Step Solvothermal Synthesis of Carbon Dots for Rapid and Accurate Determination of Hemin Content

**Yiaobo Zhang [1,†], Lin Liu [1,†], Jiahui He [1], Chengzhi Huang [1], Lei Zhan [1,*] and Chunmei Li [1,2,*]**

[1] Key Laboratory of Luminescence Analysis and Molecular Sensing (Southwest University), Ministry of Education, College of Pharmaceutical Sciences, Southwest University, Chongqing 400715, China; meowrry02@email.swu.edu.cn (Y.Z.); liul0077@163.com (L.L.); 13688192100@163.com (J.H.); chengzhi@swu.edu.cn (C.H.)

[2] NMPA Key Laboratory for Quality Monitoring of Narcotic Drugs and Psychotropic Substance, Chongqing 401121, China

* Correspondence: zhanlei6@swu.edu.cn (L.Z.); licm1024@swu.edu.cn (C.L.); Tel.: +86-2368254059 (C.L.); Fax: +86-2368367257 (C.L.)

† These authors contributed equally to this work.

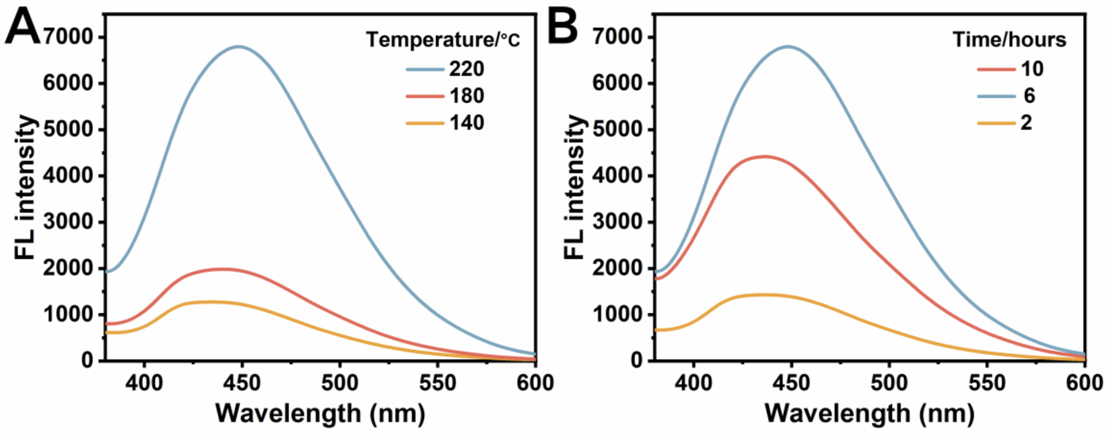


**Figure S1.** Optimization of synthesis parameters of CDs. (**A**) Fluorescence emission spectra of CDs with different synthesis temperature. (**B**) Fluorescence emission spectra of CDs with different synthesis time.

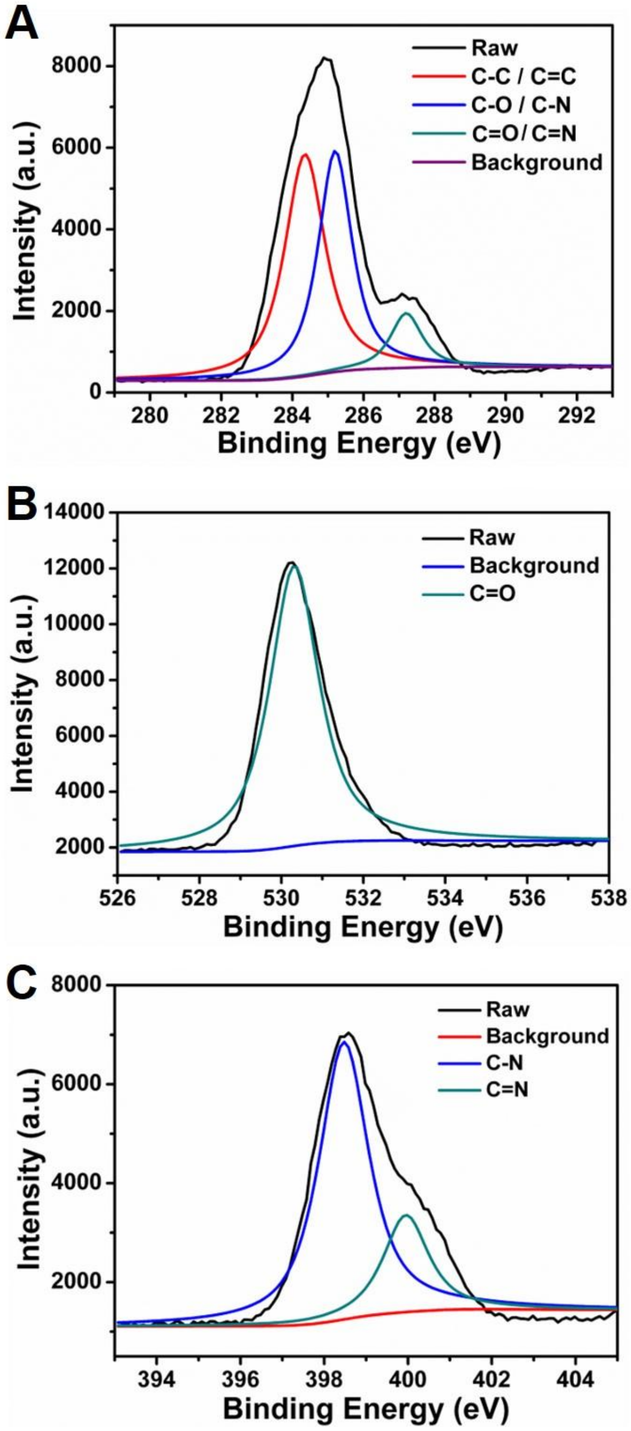


**Figure S2.** (**A**) High-resolution C 1s XPS spectrum of CDs. (**B**) High-resolution O 1s XPS spectrum of CDs. (**C**) High-resolution N 1s XPS spectrum of CDs.

**Table S1.** The influence of hemin on the fluorescence lifetime of CDs. Here, $\tau$, a and $\tau_{av}$ represented lifetime, amplitude and average lifetime, respectively. The average fluorescence lifetime was calculated according to the equation $\tau_{av} = \Sigma a_i \tau_i^2 / \Sigma a_i \tau_i$.

| sample | $\tau_1$ (ns) | $a_1$ (%) | $\tau_2$ (ns) | $a_2$ (%) | $\tau_{av}$ (ns) |
|---|---|---|---|---|---|
| CDs | 2.531 | 63.64 | 7.540 | 38.41 | 5.75 |
| CDs + hemin | 2.559 | 64.09 | 7.594 | 37.02 | 5.74 |
